# 

**Published:** 1889-03-23

**Authors:** 


CONTENTS.

**The Compulsory Elevation of Doctors** - - - - 387

**National Thrift** - - - - - - 388

**The Doctor's Pulpit.** III.—Man and His Enemies - - 389

**A Highland Laird's Family** - - - - - 390

**Treatment by Sleep and Suggestion** - - - - 391

**Shampooing versus Massage.** By Mrs. Hendley, Jeypoor, India - - - - - - 392

**The Breakdown of the Hospital System** - - - 393

**The Editor's Letter-box** - - - - - 393

**Notes and News** - - - - - - 394

**Everybody's Page** - - - - - - 396

**Annotations.**—Growing Fat—How to "Cane" Boys—Retribution - - - - - - 397

**Hints for the Sick Room.** By M. M. Basil, M.A., M.B., etc.—Bedside Manipulations - - - - 398

**False Impressions.** By Caroline Fothergill, Author of "An Enthusiast," "A Voice in the Wilderness," etc. - - 399

**Military Graveyards and Epitaphs in India**—IV. By an Old Indian - - - - - - 400

**Scraps and Gleanings—Amusements and Relaxation** 402

**EXTRA SUPPLEMENT—THE NURSING MIRROR.**

**En Passant.**—Lady Nurses—Establishing District Nurses—A Few Words to Midwives—District Nurses—Death of Miss Whately—Troubles at Stroud—Nurses as Stewardesses—Short Items - - - - - - xcvii

**Insurance for Women** - - - - - xcviii

**Nursing Journals and Amateur Editors** - - xcviii

**An Ancient House of Mercy**—Christ's Hospital, Sherburn xcix

**Everybody's Opinion.**—Nurses' Savings Fund—Lady Nurses - - - - - - - c

**Presentations, Vacancies, Notes and Queries** - c

